# Supplementary material for: Protection against lethal canine distemper virus infection by a dual epitope-targeting synthetic antibody
Source: Nat Commun. 2026 Jan 7;17:103. doi: 10.1038/s41467-025-67600-z (PMC12780139; doi:10.1038/s41467-025-67600-z)
Supplement: Supplementary file 1 — Supplementary Information [file 41467_2025_67600_MOESM1_ESM.pdf]

SUPPLEMENTARY INFORMATION FOR

Protection against lethal canine distemper virus infection by a dual epitope-targeting synthetic antibody

Melanie Scherer[1,2,#,‡], Nadia Djabeur[2,3,#], Oliver Siering[4,#], Jean-Marc Jeckelmann[3], Marianne Wyss[1], Marina Cresci[5], Morgane Di Palma Subran[5,†], Rainer Riedl[6], Patrick Chames[5], Christian K. Pfaller[4,7,8], Bevan Sawatsky[4,",*], Dimitrios Fotiadis[3,9,",*] and Philippe Plattet[1,9,",*]

[1] Division of Neurological Sciences, Vetsuisse Faculty, University of Bern, Bern, Switzerland

[2] Graduate School for Cellular and Biomedical Sciences, University of Bern, Bern, Switzerland

[3] Institute of Biochemistry and Molecular Medicine, Medical Faculty, University of Bern, Bern, Switzerland

[4] Division of Veterinary Medicine, Paul-Ehrlich-Institute, Langen, Germany

[5] Aix-Marseille Université, CNRS, INSERM, Institute Paoli-Calmettes, CRCM, Marseille, France

[6] Competence Center for Drug Discovery, ZHAW Zurich University of Applied Sciences, Institute of Chemistry and Biotechnology, Wädenswil, Switzerland

[7] Mayo Clinic Graduate School of Biomedical Sciences, Virology and Gene Therapy Track, Rochester, MN, USA

[8] Mayo Clinic, Department of Molecular Medicine, Rochester, MN, USA

[9] Multidisciplinary Center for Infectious Diseases (MCID), University of Bern, Bern, Switzerland

# These authors contributed equally

" These authors jointly supervised this work

* Corresponding authors (e-mail addresses): Dimitrios.fotiadis@unibe.ch, bevan.sawatsky@pei.de and Philippe.plattet@unibe.ch (phone number: +41 31 684 23 27)

‡ Present address: Institute of Medical Microbiology, University of Zurich, Zürich, Switzerland

† Present address: Division of Neurological Sciences, Vetsuisse Faculty, University of Bern, Bern, Switzerland

Supplementary Figure 1

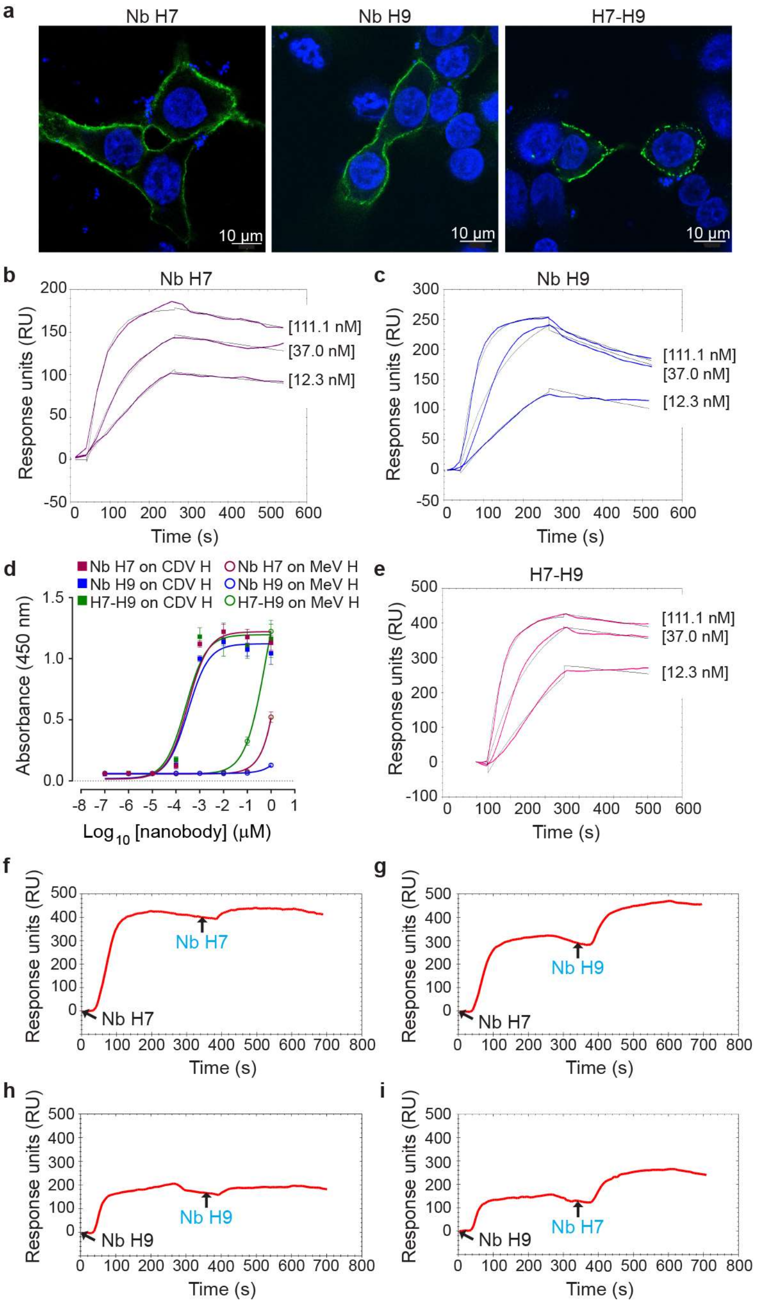

Supplementary Figure 1: Characterization of binding of Nb H7, Nb H9, and H7-H9.

(**a**) Assessment of c-Myc-tagged Nb H7, Nb H9, and H7-H9 binding to CDV H protein (from strain A75/17) by immunofluorescence analysis, using an anti-c-Myc antibody. (**b-c and e**) Binding affinity determination of Nb H7, Nb H9, and H7-H9 against immobilized solH. (**b**) Representative measurements (violet line) and 1:1 fitting (grey line) of the interaction of Nb H7 with solH. The fitted constants are association rate constant k_a = 2.18 x 10^5 ± 3.21 x 10^3 M^{-1} s^{-1}, dissociation rate constant k_d = 4.98 x 10^{-4} ± 6.79 x 10^{-5} s^{-1}, which results in dissociation constant K_D = 2.29 x 10^{-9} ± 3.46 x 10^{-10} M. (**c**) Representative measurements (blue line) and 1:1 fitting (grey line) of the interaction of Nb H9 with solH. The fitted constants are k_a = 2.05 x 10^5 ± 4.79 x 10^3 M^{-1} s^{-1}, k_d = 1.11 x 10^{-3} ± 1.21 x 10^{-4} s^{-1}, which results in K_D = 5.39 x 10^{-9} ± 7.16 x 10^{-10} M. (**d**) ELISA assays. CDV solH or MeV solH were immobilized on an ELISA plate, followed by exposure of myc-tagged nanobodies. Data are mean ± SD calculated from n = 3 independent experiments performed in technical triplicates. (**e**) Representative measurements (pink line) and 1:1 fitting (gray line) of the interaction of H7-H9 with solH. The fitted constants are k_a = 1.95 x 10^5 ± 5.60 x 10^3 M^{-1} s^{-1}, k_d = 3.64 x 10^{-4} ± 8.54 x 10^{-5} s^{-1}, which results in K_D = 1.86 x 10^{-9} ± 4.91 x 10^{-10} M. Duplicate injections of each sample were performed, and the data were fit to a 1:1 binding model within TraceDrawer software. (**f-i**) Epitope binning experiments investigated by SPR. (**f**) Upon immobilization of solH on the SPR chip, Nb H7 was first injected alone at saturating concentrations (250 nM). Subsequently a second injection was performed with Nb H7 (250 nM) mixed with Nb H7 (100 nM), or (**g**) with Nb H7 (250 nM) mixed with Nb H9 (100 nM). (**h**) Similar experimental setup as described in (f) but with the initial injection performed with Nb H9 at saturating concentrations (300 nM) and followed by the second injection containing Nb H9 (300 nM) mixed with Nb H9 (100 nM), or (**i**) Nb H9 (300 nM) mixed with Nb H7 (100 nM). The first injected Nb is indicated in black and the second one in blue. Images were generated with the TraceDrawer software.

Supplementary Figure 2

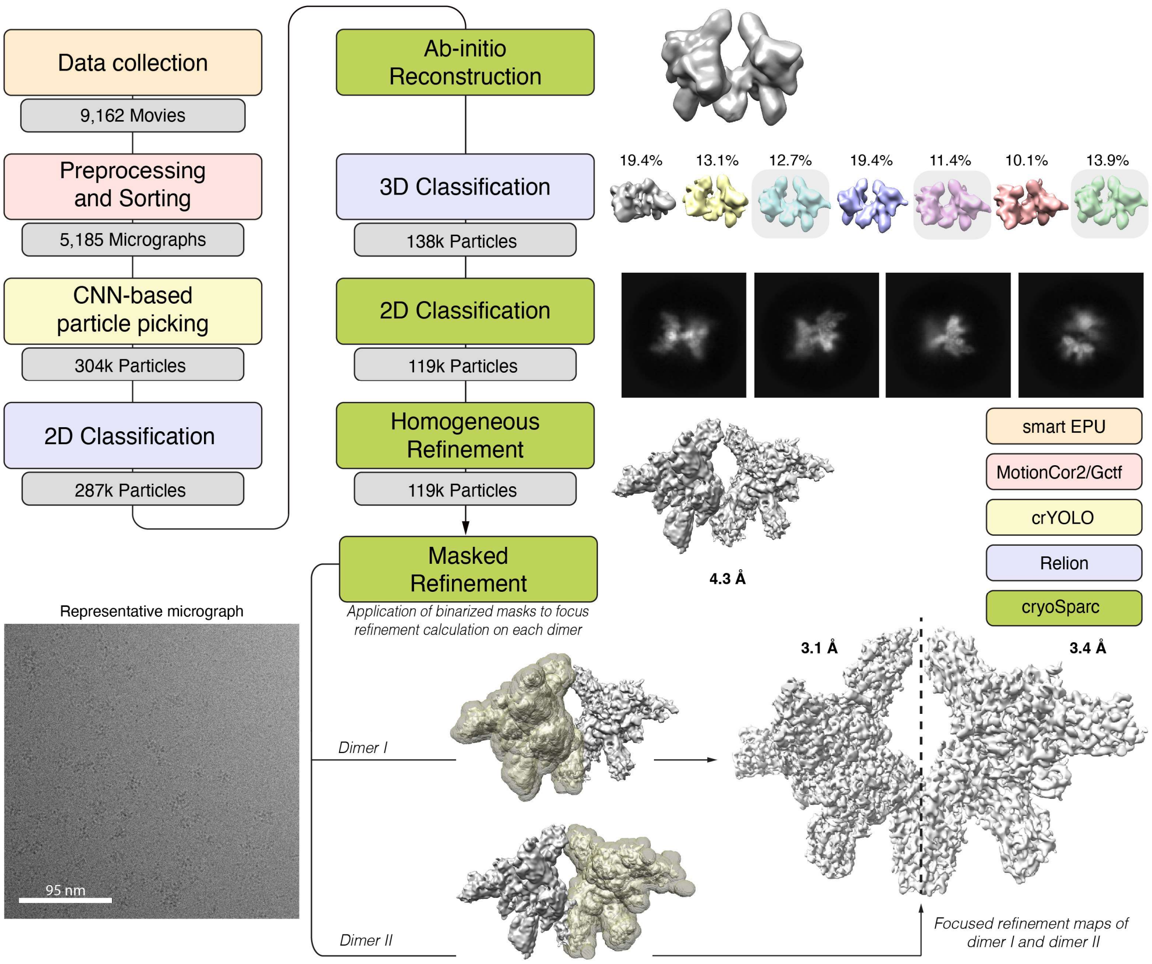

Supplementary Figure 2: Single particle cryo-EM image processing and 3D reconstruction workflow. A graphical representation of the processing steps as described in the Methods section is shown. In brief, particles from the processed micrographs (representative micrograph is displayed in the bottom-left corner) were picked using convolutional neural networks (CNN) of crYOLO. Representative 2D class averages and 3D class averages were generated within Relion (steps indicated in blue). The selected particles from the 3D classes highlighted by grey boxes were pooled and further processed, and refined in cryoSPARC (steps indicated in green). Global refinement resulted in a 4.3 Å density map of the H protein tetramer/Nbs complex. Focused refinements on individual dimers (referred to as dimer I and dimer II, see Figure 2), yielded maps at 3.1 Å and 3.4 Å, respectively. The frame-size of the shown representative 2D class averages is 352 Å. Software applied during the different steps are color-coded: smart EPU in orange, MotionCor2 and Gctf in pink, crYOLO in yellow, Relion in blue, and cryoSPARC in green.

Supplementary Figure 3

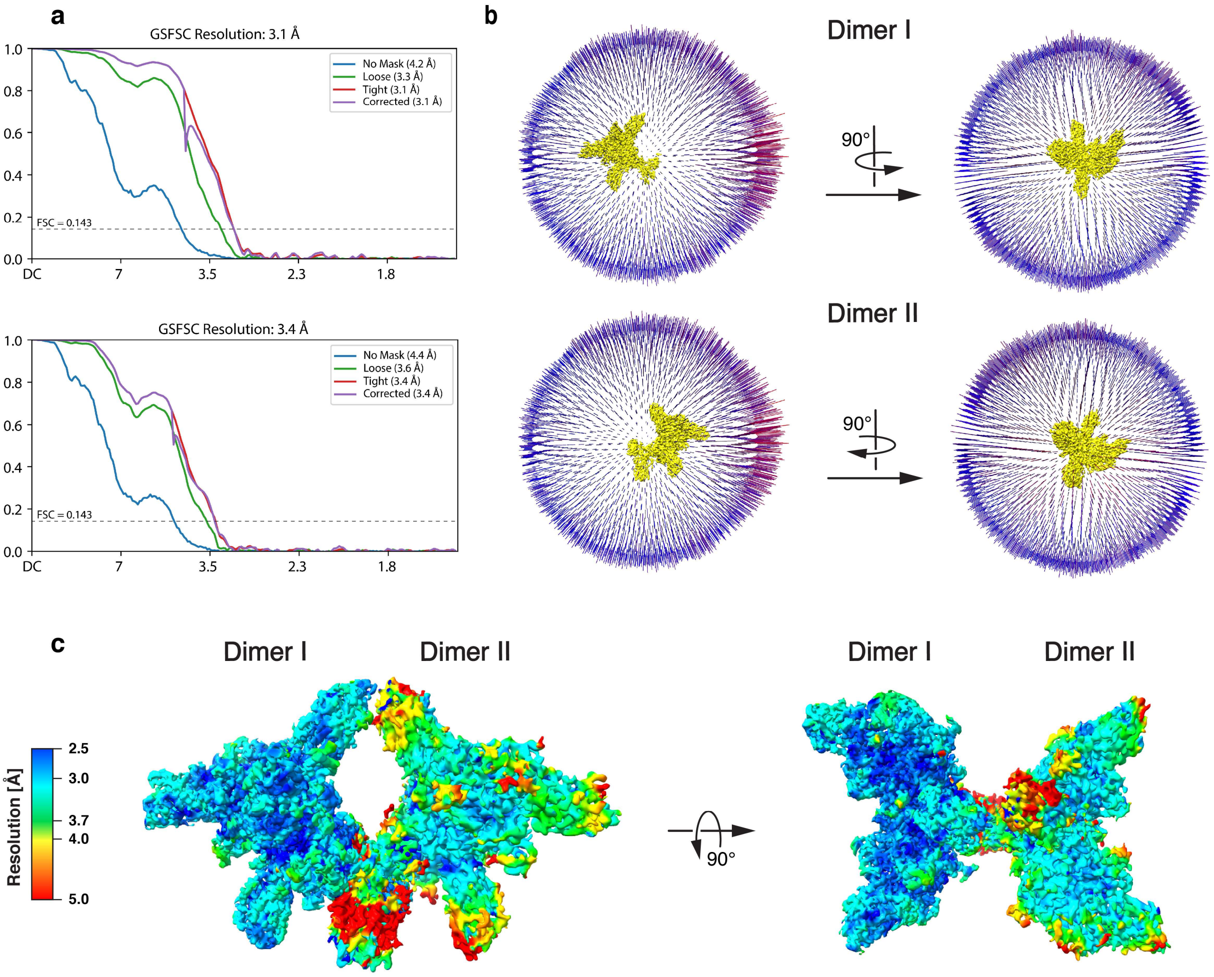

Supplementary Figure 3: Cryo-EM data analysis of CDV H protein ectodomain bound with Nbs H7 and H9.

Fourier shell correlation (FSC) plots of the two locally refined maps referring to dimer I and dimer II (**a**). The reported resolution for the maps are based on the gold-standard FSC (GSFSC) 0.143 criterion, i.e., 3.1 Å for dimer I and 3.4 Å for dimer II. (**b**) Three-dimensional representation of the angular distribution of particles included in the final 3D reconstructions of dimer I and II. (**c**) Top and side view of a combined map of the final locally refined dimer I and dimer II maps, representing the tetrameric CDV-solH ectodomain with bound Nbs. The maps are colored by the local resolution estimates calculated using cryoSPARC.

Supplementary Figure 4

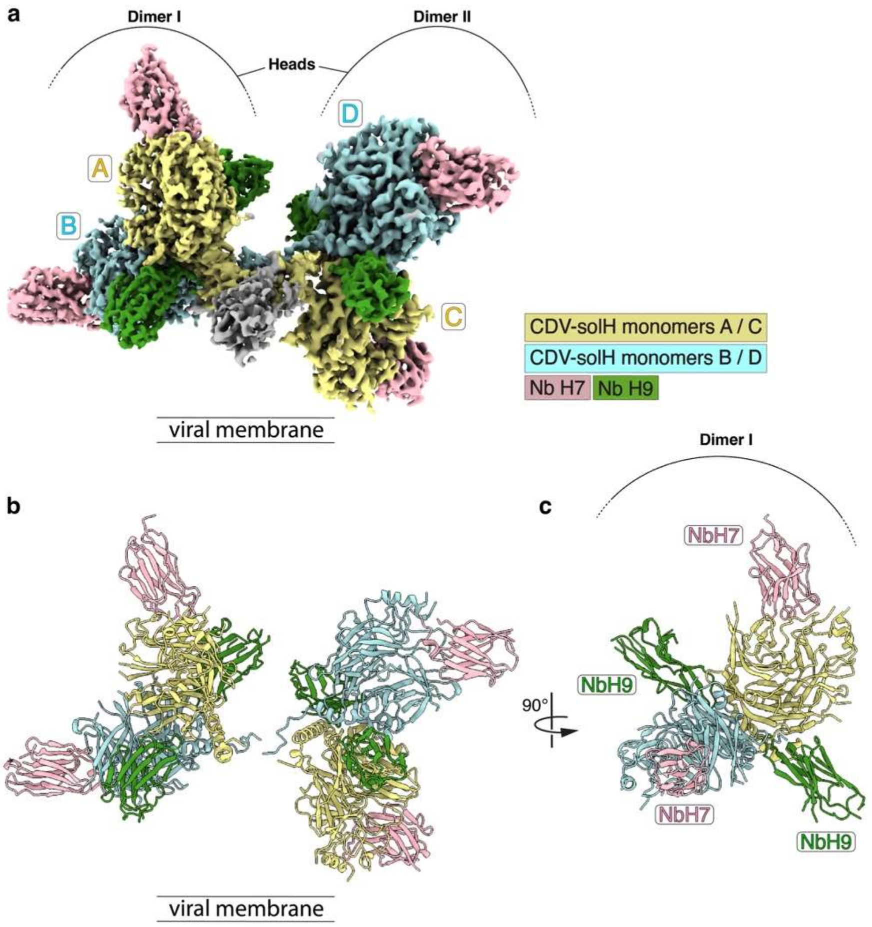

Supplementary Figure 4: Tetrameric architecture of the canine distemper virus attachment protein bound with two neutralizing nanobodies. (**a**) Full cryo-EM density map of the ectodomain of the canine distemper virus H protein (CDV-solH) with bound nanobodies H7 (pink) and H9 (green). The tetramer is organized as two head-to-head dimers (dimer I and dimer II) that are positioned above the membrane-proximal stalk (grey). The approximate location of the viral membrane is indicated. Individual H protomer densities are colored in yellow (monomers A/C) and in cyan (monomers B/D). (**b**) Model of the CDV H protein ectodomain-nanobodies complex displayed as ribbons in the same orientation as in (a). (**c**) Side-view of one head-to-head dimer (Dimer I) after a 90° rotation relative to (b).

Supplementary Figure 5

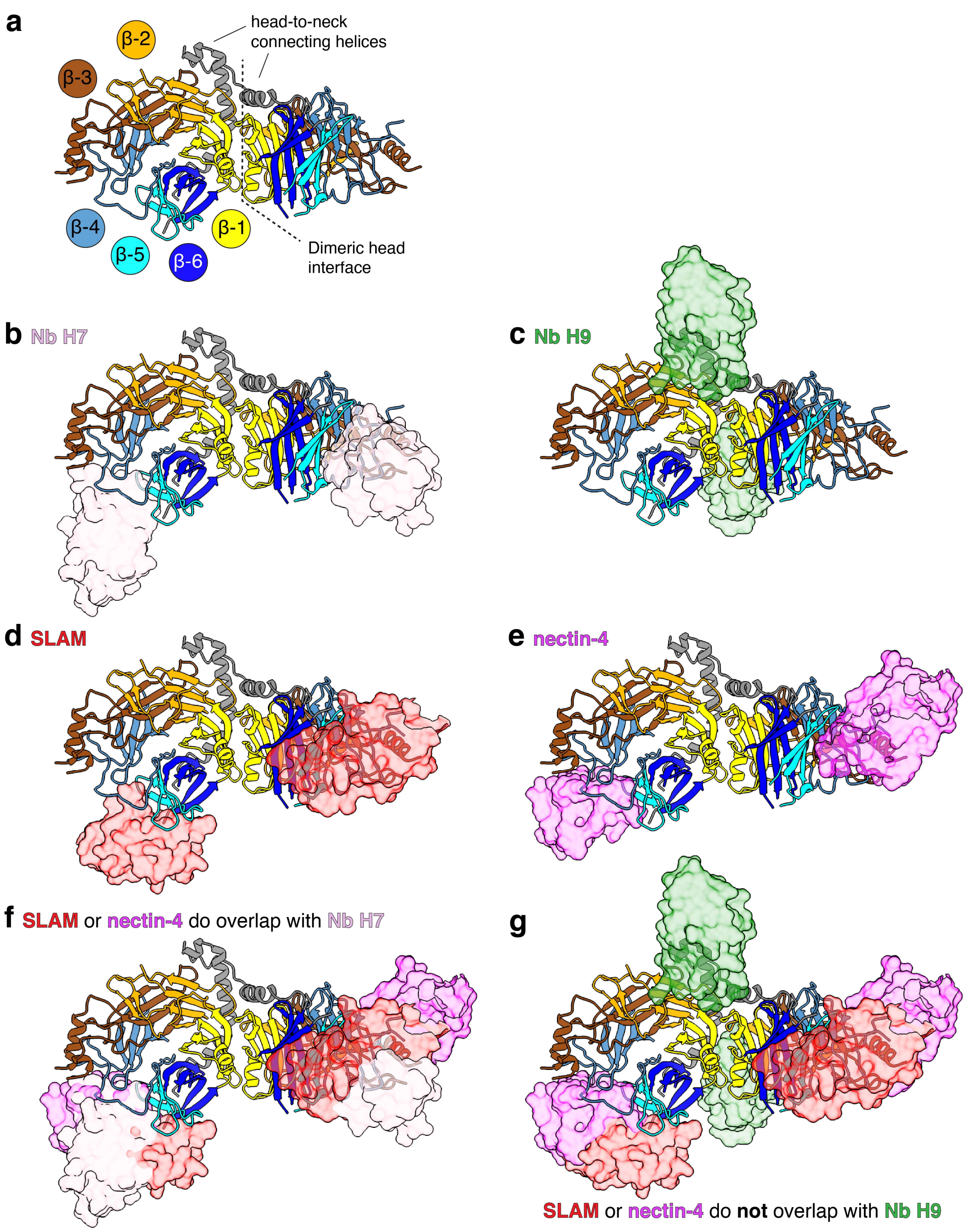

Supplementary Figure 5: Labelling of CDV H protein head domain and epitope comparison of CDV head complexes.

(**a**) Shown is the ribbon representation of a dimeric head of the CDV H protein. Whereas the dimeric head interface is indicated with a dotted line, the N-terminal α-helical region connecting the neck with the six bladed β-propeller head is colored in grey, and β-propellers in yellow (β-1), orange (β-2), brown (β-3), light blue (β-4), cyan (β-5) and blue (β-6). (**b-e**) Complexes between a dimeric CDV H-head and Nb H7 (light pink surface, panel b), Nb H9 (green surface, panel c), SLAM (red surface, panel d) and nectin-4 (magenta surface, panel e) are displayed. (**f** and **g**) Potential overlaps between SLAM, nectin-4, and Nb H7 (**f**) and Nb H9 (**g**) are shown. Note that while Nb H7 overlaps with SLAM and nectin-4, Nb H9 does not. Additionally, for both nectin-4 and SLAM, only the domains interacting with CDV H are displayed.

Supplementary Figure 6

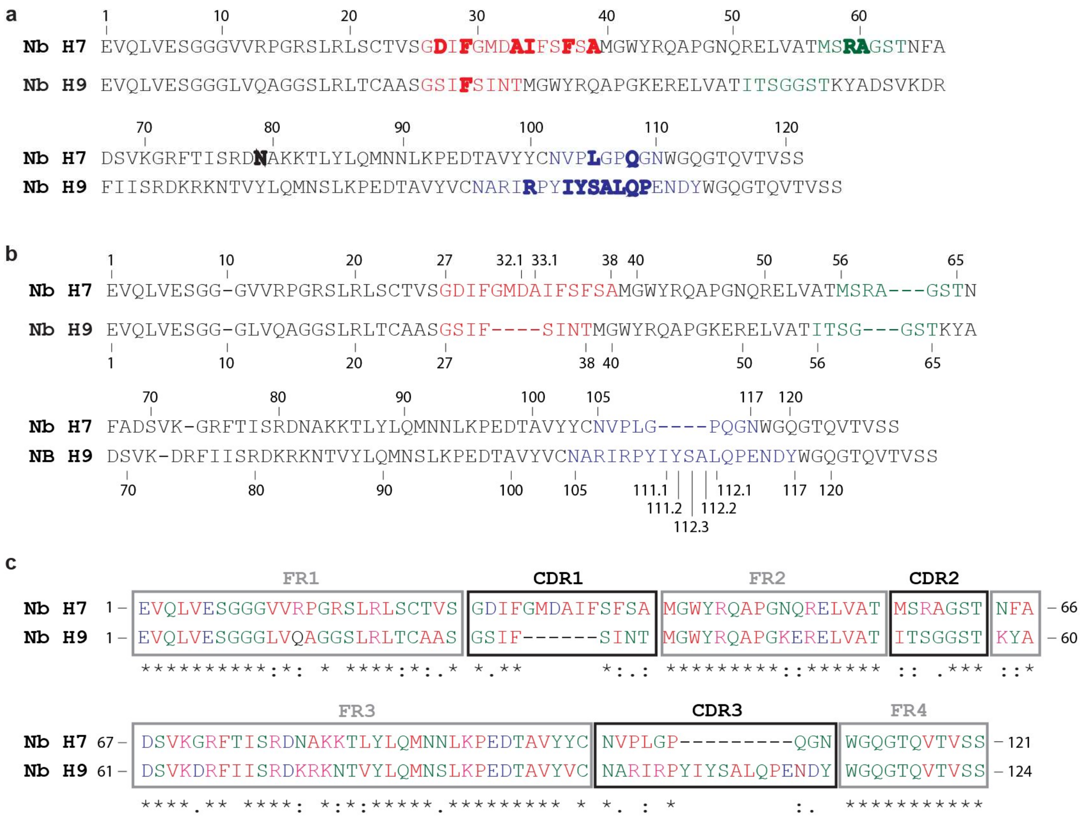

Supplementary Figure 6: Nanobodies H7 and H9 amino acid numbering and sequence comparison. (**a**) Polypeptide chain-based residue numbering. Amino acids involved in the interaction with CDV H are shown in bold. Complementarity-determining regions (CDRs) are highlighted in red (CDR1), green (CDR2), and blue (CDR3). (**b**) IMGT (ImMunoGeneTics)-based amino acid residue numbering. CDRs are color-coded as in (a). (**c**) Nbs H7 and H9 amino acid sequence alignment using the online software tool: Clustal Omega (https://www.ebi.ac.uk/jdispatcher/msa/clustalo). CDRs and framework region (FR) are shown (black and grey boxes).

Supplementary Figure 7

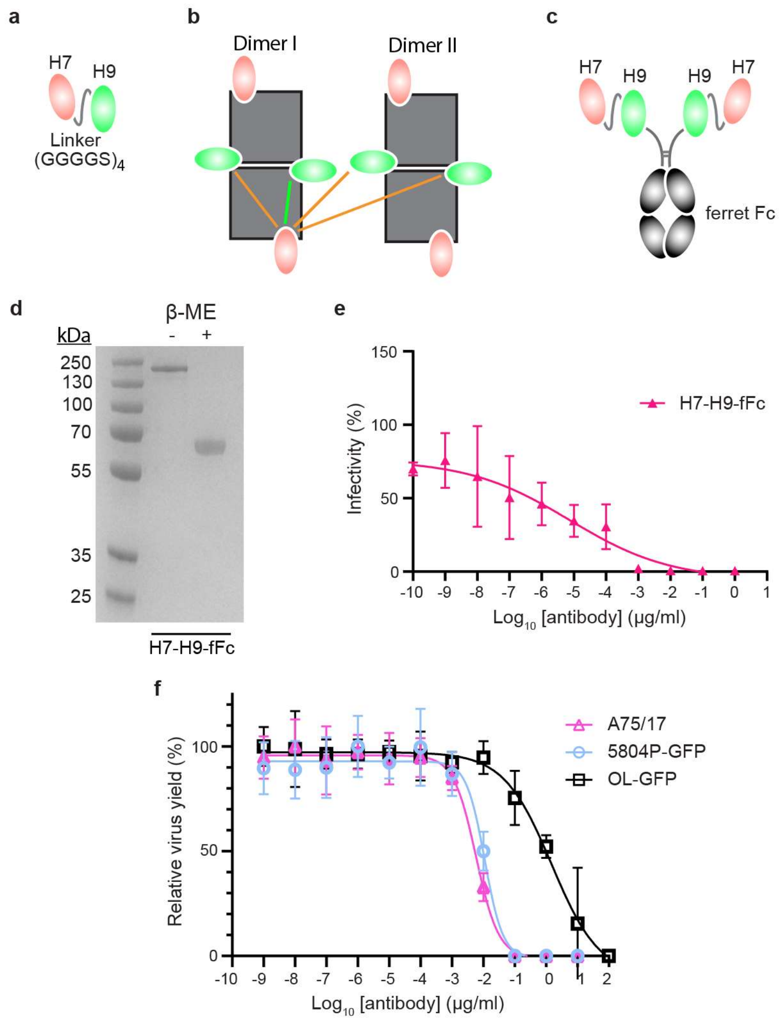

Supplementary Figure 7: Design and characterization of the tetravalent, biparatopic antibody H7-H9-fFc.

(**a**) Schematic representation of the biparatopic molecule H7-H9. Nbs H7 and H9 are color-coded in red and green, respectively. The flexible liner $(GGGGS)_4$ is indicated in gray. (**b**) Schematic representation of the four possible interactions of one biparatopic H7-H9 molecule with the CDV H protein. Both dimeric head units of the CDV H tetramer are represented as dark grey squares. The length of the linker was selected to putatively enable the simultaneous interaction of Nbs H7 and H9 to CDV H protein only with the shorter distance between two epitopes within one H head dimeric unit (green line). All other simultaneous interactions (intra and inter CDV H dimers) may not occur due to a too long distance between the two targeted epitopes (orange lines). (**c**) Schematic representation of the Nb-based antibody (H7-H9-fFc). The ferret Fc domain is highlighted in grey. (**d**) SDS-PAGE analysis of the purified H7-H9-fFc antibody. H7-H9-fFc was loaded on a gel and electrophoresis ran under reducing (β-ME+ [β-mercaptoethanol]) or nonreducing (β-ME-) conditions and stained by Instant Blue® Coomassie. (**e**) Neutralization assays against CDV A75/17$^{neon/nlucP}$. The panel depicts the relative viral infectivity at increasing concentrations of the tetravalent, biparatopic antibody H7-H9-fFc. Data are mean ± SD calculated from *n* = 3 independent experiments performed in technical triplicates. (**f**) Comparative efficacy of H7-H9-fFc against the CDV strains A75/17 and 5804P. Prior to performing *in vivo* studies in ferrets, the activity of H7-H9-fFc against 5804P was compared with that against A75/17 in a progeny virus inhibition assay using recombinant infectious versions of both strains that express GFP from an additional transcriptional cassette between the viral H and L genes. H7-H9-fFc was equally effective in its ability to neutralize both viruses, demonstrating that, in principle, 5804P is suitable for use as the challenge virus to test the efficacy of H7-H9-fFc in ferrets. Additionally, H7-H9-fFc is also active against the Onderstepoort vaccine strain CDV (OL-GFP), albeit with approximately 100-fold lower potency. Data are mean ± SD calculated from *n* = 3 independent experiments performed in technical duplicates. Curves (e and f) were plotted with GraphPad Prism v.9.

Supplementary Figure 8

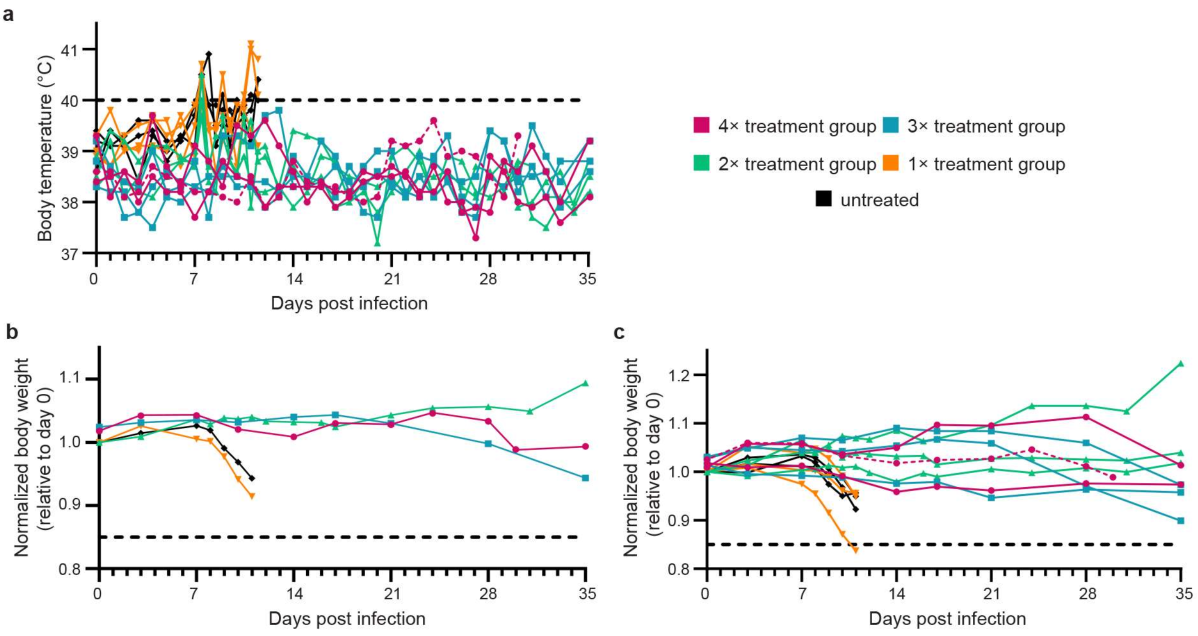

Supplementary Figure 8: Biological parameters for *in vivo* ferret studies.

(**a**) Body temperatures of individual animals. Rectal temperatures of each animal are shown as its own symbol and solid line. The dashed line at 40°C indicates the threshold for high fever. (**b**) Body weight variation. The mean weights of animals in each group (n=3) are shown as different symbols and solid lines, calculated relative to the animals' weights on day 0. (**c**) Body weight variation of individual animals. Each animal is shown with its own symbol and solid line. In (**a**) and (**c**), the purple dashed line represents one ferret in the 4× treatment group that developed neurological signs of disease and was euthanized on day 30. The dashed line (b and c) at 0.85 represents the lower weight boundary used as part of the euthanasia criteria. Each group consisted of three animals (n=3).

Supplementary Figure 9

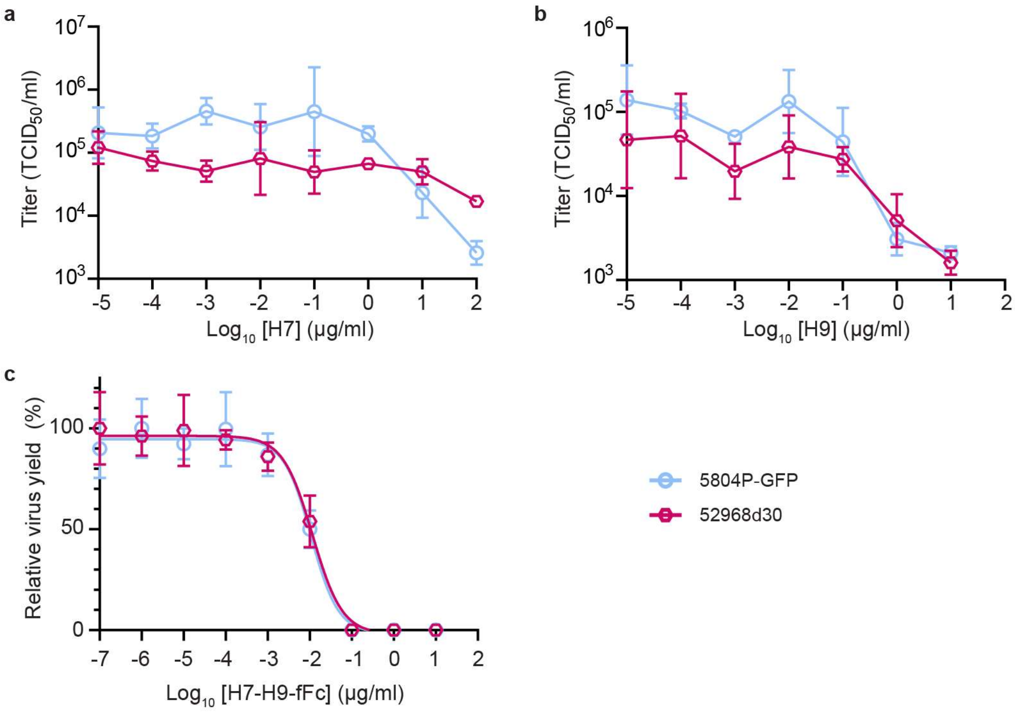

Supplementary Figure 9: Characterization of the viral isolate from the 4× treatment group ferret that developed neurological disease.

(**a**) Comparative efficacy of Nb H7 against 5804P and the day 30 viral isolate. Nb H7 had neutralizing activity against 5804P, but activity against the day 30 virus isolate was diminished. This indicates that Nb H7 binds more weakly to the day 30 isolate H protein compared to the 5804P H protein, but it was not completely abolished at the highest concentration that was used (100 µg/ml). Data are mean ± SD calculated from *n* = 3 independent experiments performed in technical duplicates. (**b**) Comparative efficacy of Nb H9 against 5804P and the day 30 viral isolate. There was no discernable difference in the ability of Nb H9 to neutralize 5804P or the day 30 isolate, indicating that binding to the H9 epitope is not substantially affected. Data are mean ± SD calculated from *n* = 3 independent experiments performed in technical duplicates. (**c**) Comparative efficacy of H7-H9-fFc against the input CDV 5804P strain (5804P-GFP) and the viral isolate harvested from ferret 52968 on day 30 (52968d30), after the onset of neurological disease (ataxia, lack of hind limb coordination, and difficulty walking). There was no substantial difference between the neutralization activity against both, demonstrating that the S546Y mutation in this isolate does not confer enhanced resistance to neutralization by H7-H9-fFc. Data are mean ± SD calculated from *n* = 3 independent experiments performed in technical duplicates. Curves were plotted using (a and b) Microsoft Excel or (c) GraphPad Prism v.9.
