# Supplementary material for: Protection against lethal canine distemper virus infection by a dual epitope-targeting synthetic antibody
Source: Nat Commun. 2026 Jan 7;17:103. doi: 10.1038/s41467-025-67600-z (PMC12780139; doi:10.1038/s41467-025-67600-z)
Supplement: Supplementary file 2 — Description of Additional Supplementary Files [file 41467_2025_67600_MOESM2_ESM.pdf]

Description of Additional Supplementary Files

Supplementary Data 1: **Polar and hydrophobic interactions in CDV-H/Nb H7 and CDV-H/Nb H9.**

CDR: complementary-determining region; FR: framework region. When amino acid residues (one letter code) are interacting with their main-chain carbonyl oxygen or nitrogen atom, then they are additionally labeled with (O) or (N).

Supplementary Data 2: **Comparison of Nb H7-targeted epitope between different CDV and MeV strains.**

Analyzed CDV and MeV strains (GenBank accession number); AM2: America 2 (AAD49703.1); AM1: America 1 (AAK54669.1); AS1: Asia 1 (BAM15593.1); AS2: Asia 2 (BAF03635.1); EU: Europe (ABF55673.1); EW: European Wildlife (ABB51156.1); EU/SAM1: Europe / South America 1 (Z47761.1); SAM2: South America 2 (ACJ23367.1); SAM 3: South America 3 (AHZ01287.1); Africa (ACS36508.1); Arctic (AGS58503.1); MeV: Measles virus IC-B (NP_056923.1), Edmonston (AAA75500.1). aa mutations are highlighted in red. # aa: amino acids.

Supplementary Data 3: **Comparison of Nb H9-targeted epitope between different CDV and MeV strains.**

Analyzed CDV and MeV strains (GenBank accession number); AM2: America 2 (AAD49703.1); AM1: America 1 (AAK54669.1); AS1: Asia 1 (BAM15593.1); AS2: Asia 2 (BAF03635.1); EU: Europe (ABF55673.1); EW: European Wildlife (ABB51156.1); EU/SAM1: Europe / South America 1 (Z47761.1); SAM2: South America 2 (ACJ23367.1); SAM 3: South America 3 (AHZ01287.1); Africa (ACS36508.1); Arctic (AGS58503.1); MeV: Measles virus IC-B (NP_056923.1), Edmonston (AAA75500.1). aa mutations are highlighted in red. # aa: amino acids.

Supplementary Data 4: **Amino acid sequence identities of H protein of different CDV strains.**

Analyzed CDV strains (GenBank accession number); AM2: America 2 (AAD49703.1); AM1: America 1 (AAK54669.1); AS1: Asia 1 (BAM15593.1); AS2: Asia 2 (BAF03635.1); EU: Europe (ABF55673.1); EW: European Wildlife (ABB51156.1); EU/SAM1: Europe / South America 1 (Z47761.1); SAM2: South America 2 (ACJ23367.1); SAM 3: South America 3 (AHZ01287.1); Africa (ACS36508.1); Arctic (AGS58503.1).

Supplementary Data 5: **Amino acid sequence identities of H protein of different morbilliviruses.**

Analyzed morbillivirus strains (GenBank accession number); CDV: Canine distemper virus (AAD49703.1); MeV: Measles virus (NP_056923.1); PPRV: Peste-des-petits-ruminants virus (CAJ01700.1); RPV: Rinderpest virus (QJD08996.1); PDV: Phocine distemper virus (BAA01207.1); CeMV: Cetacean morbillivirus (AYR16898.1); FeMV: FeMV: Feline morbillivirus (WKD80539.1).

Supplementary Data 6: Cryo-EM data collection, refinement and validation statistics.

Abbreviations: eer, electron-event representation; NAG, N-acetylglucosamine.

Supplementary Data 7: Impact of selected mutations in the CDV H protein on Nb H7, H9 and H7-H9.

Quantitative cell-to-cell fusion inhibition assays**.** The indicated Nbs were added four hours post transfection of Vero cells with plasmids encoding the CDV H protein (wt or mutant), the F protein, and LgBiT-GFP. In parallel, Vero-cSLAM cells were transfected with plasmid expressing HiBiT-RFP. Eighteen hours post cell population-mixing, cell-cell fusion was indirectly measured by recording luciferase activity. Data are mean ± SD calculated from at least n = 3 independent experiments performed in technical triplicates. Statistical significance was determined using a one-way ANOVA test, using GraphPad Prism v.10.5 (**** P≤ 0.0001; *** P≤ 0.001; ** P≤ 0.01; * P≤ 0.05; ns: non-significant). The CDV H mutants highlighted in color were also tested against the biparatopic H7-H9 construct.
