# Supplementary material for: Protection against lethal canine distemper virus infection by a dual epitope-targeting synthetic antibody
Source: Nat Commun. 2026 Jan 7;17:103. doi: 10.1038/s41467-025-67600-z (PMC12780139; doi:10.1038/s41467-025-67600-z)
Supplement: Supplementary file 4 — Reporting Summary [file 41467_2025_67600_MOESM4_ESM.pdf]

Corresponding author(s): Philippe Plattet
Last updated by author(s): Oct 21, 2025

Reporting Summary

Nature Portfolio wishes to improve the reproducibility of the work that we publish. This form provides structure for consistency and transparency in reporting. For further information on Nature Portfolio policies, see our Editorial Policies and the Editorial Policy Checklist.

Statistics

For all statistical analyses, confirm that the following items are present in the figure legend, table legend, main text, or Methods section.

n/a	Confirmed	
☐	☒	The exact sample size (*n*) for each experimental group/condition, given as a discrete number and unit of measurement
☐	☒	A statement on whether measurements were taken from distinct samples or whether the same sample was measured repeatedly
☐	☒	The statistical test(s) used AND whether they are one- or two-sided<br>*Only common tests should be described solely by name; describe more complex techniques in the Methods section.*
☒	☐	A description of all covariates tested
☐	☒	A description of any assumptions or corrections, such as tests of normality and adjustment for multiple comparisons
☐	☒	A full description of the statistical parameters including central tendency (e.g. means) or other basic estimates (e.g. regression coefficient) AND variation (e.g. standard deviation) or associated estimates of uncertainty (e.g. confidence intervals)
☐	☒	For null hypothesis testing, the test statistic (e.g. *F*, *t*, *r*) with confidence intervals, effect sizes, degrees of freedom and *P* value noted<br>*Give P values as exact values whenever suitable.*
☒	☐	For Bayesian analysis, information on the choice of priors and Markov chain Monte Carlo settings
☒	☐	For hierarchical and complex designs, identification of the appropriate level for tests and full reporting of outcomes
☒	☐	Estimates of effect sizes (e.g. Cohen's *d*, Pearson's *r*), indicating how they were calculated

Our web collection on statistics for biologists contains articles on many of the points above.

Software and code

Policy information about availability of computer code

Data collection	Cryo-EM data were collected on a Titan Krios microscope with the SerialEM software package.
Data analysis	Curves were generated with GraphPad Prism v9.5.0 and Windows Excel; Protein purification was monitored by AKTA go chromatography system using UNICORN software version 7.5 (Cyivia); SPR data were collected using OpenSPR4.0 (Nicoya); SPR data were analyzed using software TraceDrawer 1.9.1 (Ridgeview Instruments AB); ELISA data were obtained using Cytation 5 with software Gen5 3.16 (BioTek); Neutralization and quantitative cell-cell fusion data were obtained using Cytation 5 with software Gen5 3.16 (BioTek); Qualitative cell-cell fusion data was monitored using microscope EVOS M5000 (Invitrogen); Immunofluorescence Pictures were taken using Fluoview 538 FV3000 Confocal Laser Scanning Microscope (Olympus); Immunofluorescence Pictures were anlayzed and composed using software Fiji (imagej.net); SDS-PAGE picture was obtained using Gel Documentation Imager Quantum (Vilbert) with Software ST5 Xpress v16.08g; Cryo-EM image processing, structure determination and analyses were performed using standard software packages including Relion v4, Motioncorr2 v1.4, Gctf v1.6, crYOLO v1.5, cryoSPARC v4.2, Coot v0.9.8.5, Phenix v1.20, MolProbity and ChimeraX v1.7.1.

For manuscripts utilizing custom algorithms or software that are central to the research but not yet described in published literature, software must be made available to editors and reviewers. We strongly encourage code deposition in a community repository (e.g. GitHub). See the Nature Portfolio guidelines for submitting code & software for further information.

Data

Policy information about availability of data

All manuscripts must include a data availability statement. This statement should provide the following information, where applicable:

- Accession codes, unique identifiers, or web links for publicly available datasets
- A description of any restrictions on data availability
- For clinical datasets or third party data, please ensure that the statement adheres to our policy

The amino acid sequences of the 2 neutralizing single-domain antibodies are available in Extended Data Fig. 5. Cryo-EM maps and structural coordinates have been deposited into the EM database (EMDB) and Protein Data Bank (PDB) with the accession codes EMD-52024 (EMD) and 9HBP (PDB).

Research involving human participants, their data, or biological material

Policy information about studies with human participants or human data. See also policy information about sex, gender (identity/presentation), and sexual orientation and race, ethnicity and racism.

Reporting on sex and gender	*Use the terms sex (biological attribute) and gender (shaped by social and cultural circumstances) carefully in order to avoid confusing both terms. Indicate if findings apply to only one sex or gender; describe whether sex and gender were considered in study design; whether sex and/or gender was determined based on self-reporting or assigned and methods used.*<br>*Provide in the source data disaggregated sex and gender data, where this information has been collected, and if consent has been obtained for sharing of individual-level data; provide overall numbers in this Reporting Summary. Please state if this information has not been collected.*<br>*Report sex- and gender-based analyses where performed, justify reasons for lack of sex- and gender-based analysis.*
Reporting on race, ethnicity, or other socially relevant groupings	*Please specify the socially constructed or socially relevant categorization variable(s) used in your manuscript and explain why they were used. Please note that such variables should not be used as proxies for other socially constructed/relevant variables (for example, race or ethnicity should not be used as a proxy for socioeconomic status).*<br>*Provide clear definitions of the relevant terms used, how they were provided (by the participants/respondents, the researchers, or third parties), and the method(s) used to classify people into the different categories (e.g. self-report, census or administrative data, social media data, etc.)*<br>*Please provide details about how you controlled for confounding variables in your analyses.*
Population characteristics	*Describe the covariate-relevant population characteristics of the human research participants (e.g. age, genotypic information, past and current diagnosis and treatment categories). If you filled out the behavioural & social sciences study design questions and have nothing to add here, write "See above."*
Recruitment	*Describe how participants were recruited. Outline any potential self-selection bias or other biases that may be present and how these are likely to impact results.*
Ethics oversight	*Identify the organization(s) that approved the study protocol.*

Note that full information on the approval of the study protocol must also be provided in the manuscript.

Field-specific reporting

Please select the one below that is the best fit for your research. If you are not sure, read the appropriate sections before making your selection.

☒ Life sciences ☐ Behavioural & social sciences ☐ Ecological, evolutionary & environmental sciences

For a reference copy of the document with all sections, see nature.com/documents/nr-reporting-summary-flat.pdf

Life sciences study design

All studies must disclose on these points even when the disclosure is negative.

Sample size	Sample sizes for ferret experiments (n=3) were determined to provide sufficient statistical power while reducing the number of animals used in the studies according to 3R principles.<br>Sample size, i.e., number of movies recorded, was determined by maximum collection rate and the allocated time slot on the microscope.
Data exclusions	Electron micrographs were processed in Relion v4 to exclude low-quality data (e.g., images with high drift or astigmatism).
Replication	The data generated by the ferret experiment are based on sufficient animals (n=3 per group) and encompass a wide range of potential biological variability. Replication of these studies was therefore determined to not be warranted, in keeping with 3R principles.
Randomization	Ferrets were randomly assigned to experimental groups (n=3, one males and two females).
Blinding	Animal support staff and caretakers were blinded. Investigators were not blinded due to limitations in personnel who are authorized and trained to perform ferret studies.

Reporting for specific materials, systems and methods

We require information from authors about some types of materials, experimental systems and methods used in many studies. Here, indicate whether each material, system or method listed is relevant to your study. If you are not sure if a list item applies to your research, read the appropriate section before selecting a response.

Materials & experimental systems

n/a	Involved in the study
☐	☒ Antibodies
☐	☒ Eukaryotic cell lines
☒	☐ Palaeontology and archaeology
☐	☒ Animals and other organisms
☒	☐ Clinical data
☒	☐ Dual use research of concern
☒	☐ Plants

Methods

n/a	Involved in the study
☒	☐ ChIP-seq
☒	☐ Flow cytometry
☒	☐ MRI-based neuroimaging

Antibodies

Antibodies used	Mouse anti-His Antibody (Novagen), Sigma-Aldrich, Cat.# 70796-M, 1:1000 dilution<br>Peroxidase AffiniPure™ Goat Anti-Mouse IgG (H+L), Jackson ImmunoResearch, Cat.# 115-035-003, 1:5000 dilution<br>Goat anti-Mouse IgG (H+L) Cross-Adsorbed Secondary Antibody, Alexa Fluor™ 647, Invitrogen, Cat.# A-21235, 1:5000 dilution<br>Mouse anti c-Myc Monoclonal Antibody (9E10), Invitrogen, Cat.# MA1980, diluted 1:500<br>Goat anti-Mouse IgG (H+L) Cross-Adsorbed Secondary Antibody, Alexa Fluor™ 488, Invitrogen, Cat.# A11001, diluted 1:1000<br>THE™ NWSHPQFEK Tag Antibody, mAb, Mouse, GenScript, Cat.# A01732, diluted 1:5000<br>Goat Anti-Mouse Immunoglobulins/HRP (affinity isolated), Agilent, Cat.# P0447, diluted 1:3000<br>Anti-Human IgG (Fc specific)–Peroxidase antibody produced in goat, Sigma-Aldrich, Cat.# A0170, diluted 1:10000
Validation	All antibodies were bought from commercial vendors and validated by the manufacturers.

Eukaryotic cell lines

Policy information about cell lines and Sex and Gender in Research

Cell line source(s)	HEK-293T/17 (ATCC, CRL-11268), ExpiCHO-S (Thermo Fisher Scientific, A29127), Vero (ATCC, CCL1-81), Vero cells stably expressing canine SLAM/CD150 (Vero-cSLAM; von Messling et al, 2003, J Virol 77:12579-12591 and Tatsuo et al, 2001 J Virol 75(13):5842-50.
Authentication	HEK-293T/17, ExpiCHO-S and Vero cells were purchased commercially and were not further validated. Vero-cSLAM cells were authenticated by Western blotting for SLAM expression (detection via C-terminal HA-tag) and by susceptibility to CDV infection.
Mycoplasma contamination	Cells were negative for Mycoplasma contamination via PCR.
Commonly misidentified lines (See ICLAC register)	No commonly misidentified cell lines were used.

Animals and other research organisms

Policy information about studies involving animals; ARRIVE guidelines recommended for reporting animal research, and Sex and Gender in Research

Laboratory animals	The studies used male and female ferrets (Mustela putorius furo) at least 16 weeks of age. This is stated in the Materials and Methods.<br><br>A llama glama (~2 years old, male) was used for immunization.
Wild animals	No wild animals were used.
Reporting on sex	Males and female ferrets were used to account for differences in biological responses between the sexes.
Field-collected samples	This study did not used field-collected samples.
Ethics oversight	Ferret experiments with CDV were conducted in the animals BSL-2 facility of the Paul-Ehrlich-Institute in accordance with the German Animal Welfare Law (Tierschutzgesetz) and all applicable German and European regulations under protocol number V54-19c20/15-F107/1024.

Llama immunizations were executed in strict accordance with good animal practices, following the EU animal welfare legislation law and were approved by local authorities (French Ministry of Higher Education for Research and Innovation).

Note that full information on the approval of the study protocol must also be provided in the manuscript.

Plants

Seed stocks

Report on the source of all seed stocks or other plant material used. If applicable, state the seed stock centre and catalogue number. If plant specimens were collected from the field, describe the collection location, date and sampling procedures.

Novel plant genotypes

Describe the methods by which all novel plant genotypes were produced. This includes those generated by transgenic approaches, gene editing, chemical/radiation-based mutagenesis and hybridization. For transgenic lines, describe the transformation method, the number of independent lines analyzed and the generation upon which experiments were performed. For gene-edited lines, describe the editor used, the endogenous sequence targeted for editing, the targeting guide RNA sequence (if applicable) and how the editor was applied.

Authentication

Describe any authentication procedures for each seed stock used or novel genotype generated. Describe any experiments used to assess the effect of a mutation and, where applicable, how potential secondary effects (e.g. second site T-DNA insertions, mosiacism, off-target gene editing) were examined.
